# The Germania

**Published:** 1889-08

**Authors:** 


### BOOK NOTICES.

*The Germania* is a fortnightly journal for the study of the German language and literature. It contains a series of German exercises, well adapted for those who would acquire a good knowledge of the language. Subscription price, $3.00 per year. Address GERMANIA, P. O. Box 90, Manchester, N. H.

